# Nobiletin Inhibits Cell Viability *via* the SRC/AKT/STAT3/YY1AP1 Pathway in Human Renal Carcinoma Cells

**DOI:** 10.3389/fphar.2019.00690

**Published:** 2019-07-09

**Authors:** Di Wei, Geng Zhang, Zheng Zhu, Yu Zheng, Fei Yan, Chongxian Pan, Zhiyong Wang, Xian Li, Fuli Wang, Ping Meng, Wanxiang Zheng, Zhao Yan, Dongsheng Zhai, Zifan Lu, Jianlin Yuan

**Affiliations:** ^1^Department of Urology, Xijing Hospital, Fourth Military Medical University, Xi’an, China; ^2^Division of Hematology and Oncology, Department of Internal Medicine, School of Medicine, University of California, Davis, Sacramento, CA, United States; ^3^Department of Orthopaedics, Xijing Hospital, Fourth Military Medical University, Xi’an, China; ^4^State Key Laboratory of Cancer Biology, Department of Pharmacogenomics, Fourth Military Medical University, Xi’an, China

**Keywords:** nobiletin, renal carcinoma cells, SRC, AKT, STAT3, YY1AP1

## Abstract

Nobiletin is a polymethoxy flavonoid isolated from *Citrus depressa* and *Citrus reticulata*. It has been reported that nobiletin can suppress tumors. We primarily explored the antitumor effects of nobiletin and the associated potential mechanisms in ACHN and Caki-2 renal carcinoma cells. A CCK-8 assay and cloning experiments were used to assess cell viability, and a transwell assay and scratch test were used to assess metastatic ability. The cell cycle was analyzed by flow cytometry, whereas apoptosis was analyzed using flow cytometry and a terminal dexynucleotidyl transferase (TdT)-mediated dUTP nick end labeling (TUNEL) assay. Protein expression was examined by Western blot and immunofluorescence. Renal cancer cells were subcutaneously transplanted into nude mice for *in vivo* studies. The data showed that nobiletin administration significantly dose- and time-dependently suppressed renal cancer cell proliferation; moreover, nobiletin treatment induced cell cycle arrest in the G0/G1 phase and promoted apoptosis. Immunofluorescence analysis indicated that nobiletin decreased the nuclear localization of signal transducer and activator of transcription 3 (STAT3) and YY1-associated protein 1 (YY1AP1). Western blot showed that the levels of phosphorylated SRC, phosphorylated AKT serine/threonine kinase (AKT), and phosphorylated STAT3 were decreased, whereas that of phosphorylated YY1AP1 was increased. The results further showed that application of insulin-like growth factor 1 (IGF1) was able to reverse the nobiletin-induced changes in the levels of phosphorylated AKT, phosphorylated STAT3, and phosphorylated YY1AP1, and could also reverse the antitumor effects of nobiletin. The results of *in vivo* experiments showed that, compared to the control, tumor volume and weight were both reduced following nobiletin treatment. In conclusion, our study demonstrated that nobiletin can inhibit renal carcinoma cell viability and provides a novel therapeutic approach for the treatment of kidney cancer.

## Introduction

Renal cell carcinoma is the most common type of kidney cancer, arising mostly from renal tubular epithelial cells and accounting for more than 90% of all renal tumors (Global Burden of Disease Cancer et al., 2015; Torre et al., 2015; Hsieh et al., 2017; Siegel et al., 2018; Zhao et al., 2018). Kidney cancer development is influenced by numerous risk factors, such as obesity, hypertension, work-related factors, diet, lifestyle, and smoking (Chow et al., 2000; Hunt et al., 2005; Renehan et al., 2008). The current diagnostic methods for kidney cancer rely on imaging procedures like B-mode ultrasound and computed tomography, and the therapeutic options are based on surgical procedures. Since clear cell carcinoma results mainly from mutations in the Von Hippel-Lindau (*VHL*) gene that lead to increased hypoxia inducible factor 1 (HIF1) activity and angiogenesis, tyrosine kinase inhibitor (TKI) drugs that target the platelet-derived growth factor receptor (PDGFR) and vascular endothelial growth factor receptor (VEGFR) are predominantly used for adjuvant therapy. However, surgical trauma and the high price of targeted drug-based therapy constitute major challenges for patients. Another approach favors the inclusion of food with anticancer effects in the daily diet, aiming to prevent and treat kidney cancer. For example, apigenin (Perrott et al., 2017) and allicin (Borlinghaus et al., 2014) have been shown to exhibit some antitumor activity. Similarly, flavonoids, compounds that are present mainly in vegetables and citrus fruits, also exert anti-inflammatory, antiangiogenetic, and proapoptotic effects (Kohno et al., 2001; Lima et al., 2018; Ferreira de Oliveira et al., 2019).

Flavonoids are a class of mainly isomeric polyphenolic compounds, of which nobiletin, found mainly in oranges and lemons, is an important member (Nogata et al., 2006). Previous studies have shown that nobiletin can downregulate nitric oxide synthase, improve 2,4,6-trinitrobenzene sulfonate-induced colitis (Xiong et al., 2015), prevent and treat osteoporosis by inhibiting the nuclear factor-kappa B (NF-κB)-dependent synthesis of prostaglandin E1 (Harada et al., 2011), and improve cognitive ability in animal models of Alzheimer’s disease (Nakajima et al., 2015). In addition, an increasing number of studies have reported that nobiletin also has antitumor effects. Nobiletin has been reported to reduce the migration ability of liver cancer cells by inhibiting the expression of AKT and extracellular signal-regulated protein kinases (ERKs) (Shi et al., 2013). In breast cancer, nobiletin has been found to significantly inhibit the protein tyrosine kinase 2 (PTK2)/SRC/STAT3 angiogenetic signaling pathway, and, consequently, tumor proliferation (Sp et al., 2017). Moreover, nobiletin also inhibits the expression of HIF1A and AKT, thereby preventing tumor cell proliferation (Chen et al., 2015). In addition, a combination of nobiletin and chemotherapy significantly increased the efficacy of the latter, partly counteracting resistance to chemotherapy (Ma et al., 2015). All these findings demonstrate that nobiletin exerts several beneficial pharmacological activities in the human body, with a significant therapeutic effect against tumor metastasis and proliferation.

Although numerous studies have reported the antitumor effects of nobiletin, few have reported on its effects in kidney cancer. Here, we report preliminary data showing that nobiletin can inhibit the proliferation of renal carcinoma cells. To elucidate the underlying mechanisms of the antitumor effects of nobiletin, we mainly investigated the inhibitory effect of nobiletin on the proliferation of renal carcinoma cells and its interference with signal transduction pathways, and further confirmed this effect *in vivo*.

## Materials and Methods

### Reagents

Nobiletin (>99% purity) was purchased from MedChemExpress (Monmouth Junction, NY, USA); the CCK-8 solution was obtained from Dojindo Molecular Technologies, Inc. (Tokyo, Japan); fetal bovine serum was obtained from ZETA (San Francisco, CA, USA); trypsin and dimethyl sulfoxide (DMSO) were obtained from Thermo Fisher Scientific (Waltham, MA, USA); Eagle’s minimum essential medium (EMEM) and McCoy’s 5A media were obtained from Gibco (Gaithersburg, MD, USA). Antibodies against AKT, STAT3, SRC, YY1AP1, caspase 3, caspase 9, phospho-AKT, phospho-STAT3, phospho-YY1AP1, phospho-SRC, cleaved caspase 3, and cleaved caspase 9, as well as all secondary antibodies, were purchased from Cell Signaling Technology (Danvers, MA, USA). Stattic and Verteporfin were obtained from MedChemExpress. IGF1 was obtained from BD Bioscience (Shanghai, China).

### *In Vivo* Experiments

All animal experiments complied with ARRIVE guidelines and were carried out in strict accordance with the recommendations in the Guide for the Care and Use of Laboratory Animals of the National Institutes of Health (NIH Publication no. 8023, revised 1978). Specific-pathogen-free, 4-week-old male nude mice and C57 mice were purchased from Beijing Vital River Laboratory Animal Technology. The specific-pathogen-free-grade rearing environment was maintained by a trained person. Mice were housed individually in a climate-controlled room on a 12:12-h light-dark cycle (lights on, 08:00 to 20:00), with controlled temperature (22 ± 1°C) and humidity (50 ± 10%). Abundant healthy food and water were available to the mice. Approximately 5 × 10^6^ cells of the renal carcinoma cell line, ACHN, were suspended in 200 µl of PBS, followed by inoculation under the dorsal skin of the nude mice. The tumor size was recorded every 3 days, and the tumor volume was calculated according to the formula V = 0.5 × a × b^2^, where a and b denote the length and width, respectively. The experimental group was administered nobiletin *via* gastric lavage, at a dose of 40 mg/kg·day^−1^, in accordance with previous studies (Chen et al., 2015). The control group was administered the equivalent amount of physiological saline. All animals were sacrificed after 24 days, and the tumors were excised and weighed. The tumor tissues were fixed in 4% paraformaldehyde, embedded in paraffin, and cut into 5-µm-thick paraffin sections.

### Cell Culture and Handling

The renal carcinoma cell lines, ACHN and Caki-2, were purchased from the Shanghai cell bank (Shanghai, China). All cells were cultured in media containing 100 U/ml penicillin, 100 μg/ml streptomycin, and 10% fetal bovine serum (MEM for ACHN cells and McCoy’s 5A for Caki-2 cells) at 37°C in a humidified atmosphere with 5% CO_2_. Nobiletin was dissolved in DMSO to yield a 50-mM stock solution and was dissolved in culture medium to yield the working solution with 0.5% DMSO. An equal concentration of DMSO was added to the control group.

### Cell Proliferation Assay

The CCK-8 assay was used to assess cell proliferation. The cell concentration was adjusted to 3 × 10^3^ cells/well, and the cells were seeded into a 96-well plate, followed by 24 h of culture at 37°C in an atmosphere with 5% CO_2_. Different concentrations of nobiletin were subsequently added, and cultivation continued for a further 48 h. After removing the culture medium, the CCK-8 reaction solution was added according to the manufacturer’s instructions, and the absorbance was measured at 450 nm (A450). Relative cell viability was calculated *via* the A450 of the experimental group compared to that of the control group, expressed as a percentage. Each experiment was conducted in triplicate.

### Plate Colony-Forming Assay

The ACHN and Caki-2 cells were transferred into a cell suspension and seeded into six-well plates (Corning, NY, USA) at a density of 400 cells/well. After 24 h, cells were treated with different nobiletin concentrations (80 and 120 µM for ACHN cells, and 40 and 80 µM for Caki-2 cells) for 48 h. The nobiletin-containing medium was then removed and replaced by complete medium for 14 days. The cells were then fixed with 4% paraformaldehyde for 40 min and stained with 0.5% crystal violet at room temperature for 2 h.

### Invasion and Migration Assay

The scratch test was conducted according to a previously published protocol (Karakus et al., 2018). The cells were seeded into six-well plates until confluence, following which a needle was used to scratch the middle of the individual wells from top to bottom. Floating cells were removed by washing twice with PBS. Cultivation was subsequently continued for 24 h in serum-free medium, with or without nobiletin (40 µM for Caki-2 cells and 80 µM for ACHN cells).

The transwell assay was conducted according to a previously published method (Moradi Monfared et al., 2019). Matrigel (Corning NY, USA) was diluted in pre-cooled culture medium according to the manufacturer’s instructions. Subsequently, the matrigel was poured into the small chamber of a transwell plate and incubated overnight at 37°C to allow it to set. Each small transwell chamber was seeded with 1 × 10^4^ cells, and serum-free medium, with or without nobiletin (40 µM for Caki-2 cells and 80 µM for ACHN cells), was added into the upper chamber, whereas medium with 10% serum was added to the lower chamber. The cells were cultured at 37°C in a humidified atmosphere with 5% CO_2_ for 48 h. The cells below the sieve membrane were then fixed with 4% paraformaldehyde for 1 h and stained for 2 h with 0.5% crystal violet. Finally, the stained cells were counted under a microscope.

### Immunocytochemistry

The Caki-2 cells were seeded into a culture dish for laser confocal microscopy (Wuhan Guge Biotechnology Co., Ltd, China) at a density of 3 × 10^3^ cells/dish, and the experimental group was treated with nobiletin for 24 h. Subsequently, the cells were fixed with 4% paraformaldehyde for 20 min and washed three times with PBS, for 5 min each, then permeabilized with 0.2% Triton X-100 for 10 min, washed again three times with PBS, for 5 min each, and blocked with 5% Bovine Serum Albumin (BSA) (Thermor) for 1 h. The resulting samples were incubated with antibodies against STAT3 (1:1,000) and YY1AP1 (1:600) at 4°C overnight, washed three times with PBS, for 5 min each, followed by incubation with fluorescein isothiocyanate (FITC)-labeled goat anti-rabbit IgG secondary antibody (1:1,000) in the dark for 2 h. Finally, 1 µg/ml 4,6-diamino-2-phenyl indole (DAPI) was added, and the samples were incubated for 10 min, washed three times with PBS, for 5 min each, and observed under a confocal laser microscope.

### Apoptosis Assay

Cells were seeded into the wells of a six-well plate at a density of 2 × 10^5^ cells/well. After cultivation at 37°C in an atmosphere with 5% CO_2_ for 24 h, different concentrations of nobiletin were added, and the cultivation was continued for a further 48 h. The cells were subsequently trypsinized and transferred to a centrifuge tube, washed twice with PBS, and resuspended at a density of 1 × 10^6^ cells/ml in the combined buffer solution. An aliquot (100 µl) of the cell suspension (1 × 10^5^ cells) was transferred to a 5-ml tissue culture tube. Subsequently, 5 µl of FITC-labeled annexin V and 5 µl of propidium iodide (PI) was added to the cell suspension, gently vortexed, and incubated at room temperature in the dark for 15 min. Then, 400 µl of 1× combined buffer was added to each tube, and the samples analyzed *via* flow cytometry within 1 h. Each experiment was conducted in triplicate.

### TUNEL Staining

The paraffin-embedded sections were dewaxed, and antigen retrieval was performed using proteinase K (Servicebio, China). The sections were washed three times with PBS, for 5 min each. Subsequently, a membrane lysis solution (Servicebio) was added, and the sections incubated at room temperature for 20 min, followed by washing three times with PBS, for 5 min each. According to the instructions of the TUNEL assay kit (Roche, Switzerland), the sections were placed in a humidified box and incubated at 37°C for 2 h, with a small amount of water added inside the box to ensure sufficient humidity. Subsequently, the samples were washed three times with PBS, for 5 min each. After removing the PBS, DAPI staining solution was added slowly within a circle, and the samples were incubated in the dark for 10 min. To remove excess stain, the glass slides were placed in PBS (pH 7.4) and washed three times by shaking, for 5 min each. The sections were spin-dried briefly and sealed using a fluorescence-quenching slide-sealing agent and subsequently analyzed under a fluorescence microscope. For DAPI, an excitation wavelength of 330 to 380 nm and emission wavelength of 420 nm was used, whereas for FITC an excitation wavelength of 465 to 495 nm and emission wavelength of 515 to 555 nm were used.

### Immunofluorescence Staining of Tissue Sections

The paraffin-embedded sections were dewaxed, and antigen retrieval was performed using EDTA antigen retrieval buffer (Servicebio), followed by three 5-min washes with PBS (7.4). The sections were placed in a 3% hydrogen peroxide solution and incubated at room temperature in the dark for 25 min, followed by three PBS washes, for 5 min each. The sections were blocked with 5% BSA (Thermo Fisher Scientific) for 1 h. After removing the blocking solution, an anti-KI67 antibody (1:200; Servicebio) was dropped onto the surface of the section, and the glass slide was placed in a humidified box and incubated overnight at 4°C. The next day, the glass slide was washed three times with PBS, for 5 min each, and then incubated in the dark with a Cy3-labeled goat anti-rabbit IgG secondary antibody (1:1,000; Servicebio) for 2 h. Subsequently, 1 µg/ml DAPI solution was added and the section was incubated for 10 min, followed by three PBS washes, for 5 min each. The stained slides were analyzed under a fluorescence microscope. For DAPI, an excitation wavelength of 330 to 380 nm, and an emission wavelength of 420 nm were used, whereas for Cy3, an excitation wavelength of 510 to 560 nm and an emission wavelength of 590 nm were used.

### Western Blot Analysis

Cells were seeded into six-well plates, and different concentrations of nobiletin were added, followed by cultivation for 24 h. Subsequently, the cells were washed twice with PBS and then lysed using Radio-Immunoprecipitation Assay (RIPA) buffer (Cell Signaling Technology). A bicinchoninic acid protein assay kit (Thermo Fisher Scientific) was used to determine protein concentration. Protein samples were separated by electrophoresis on 10% acrylamide dodecyl sulfate,sodium salt-Polyacrylamide gel electrophoresis (SDS-PAGE) gels, transferred to a cellulose acetate membrane, and blocked for 2 h at room temperature in TBS with 0.1% Tween 20 and 5% skimmed milk. The membrane was incubated with an appropriately diluted primary antibody overnight at 4°C, washed three times with TBST, and incubated with the secondary antibody for 2 h at room temperature. Subsequently, the membrane was washed again three times with TBST, and a chemiluminescence solution (Thermo Fisher Scientific) was added to develop the bands.

### Statistical Analysis

All data were expressed as means ± standard deviation. Differences between two groups were analyzed using the *t*-test, and differences between three or more groups were analyzed using single-factor analysis of variance (one-way ANOVA) in SPSS (version 16.0 for Windows). Differences were considered statistically significant at *P* < 0.05.

## Results

### Nobiletin Inhibited the Proliferation of Renal Carcinoma Cells

**Figure 1:**
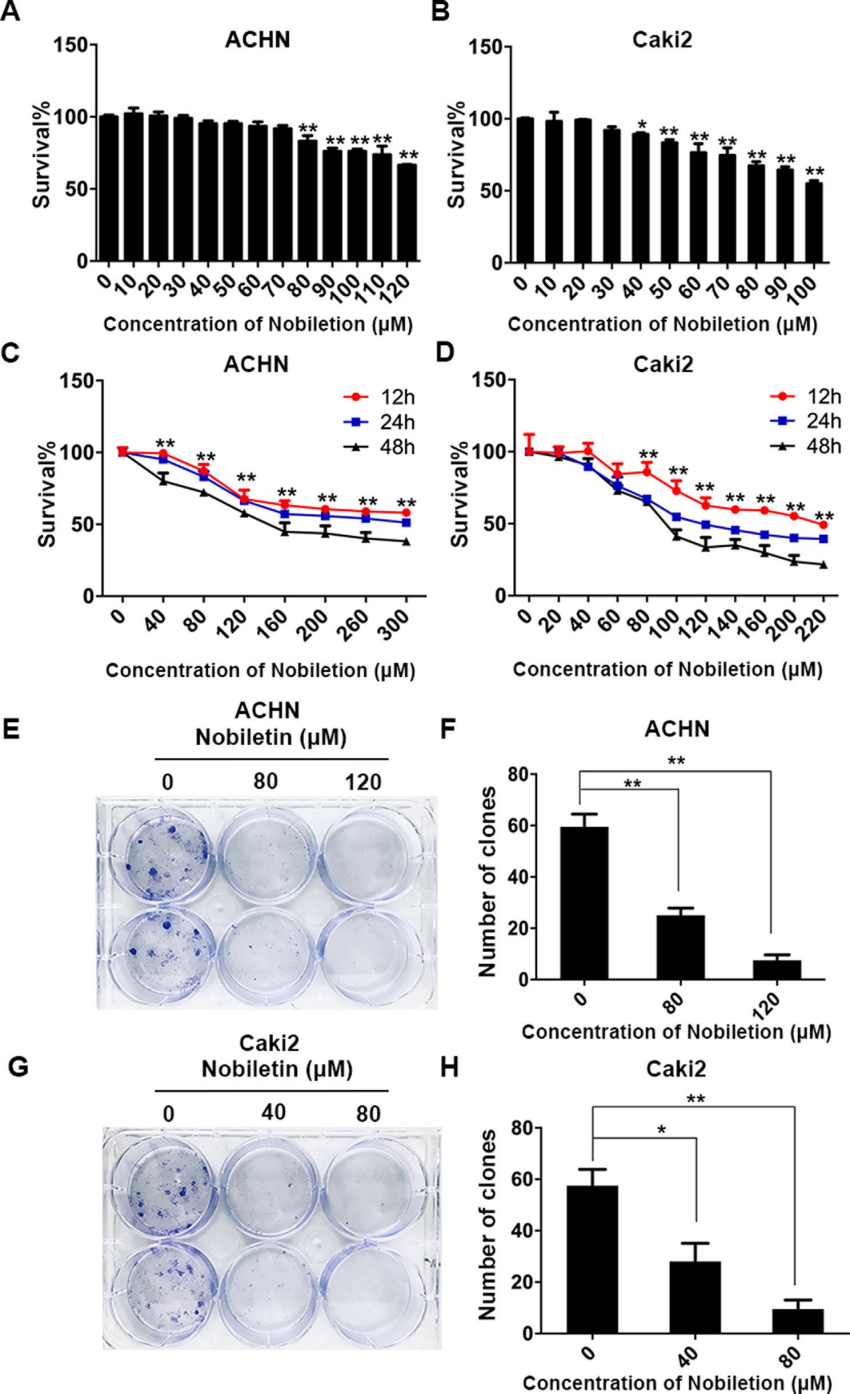
Nobiletin suppresses the proliferation of renal cancer cells. ACHN **(A)** and Caki-2 **(B)** renal carcinoma cells were treated with different concentrations of nobiletin for 24 h, and then the inhibition rates were examined. ACHN **(C)** and Caki-2 **(D)** cells were treated with different concentrations of nobiletin for 12, 24, and 48 h, and then the inhibition rates were examined. Colony-formation assay of ACHN cells **(E)** and the number of colonies are shown in the graph **(F)**. Colony-formation assay of Caki-2 cells **(G)** and the number of colonies are shown in the graph **(H)**. Data are presented as means ± SD. *P < 0.05, **P < 0.01, as compared to control.

The ACHN and Caki-2 renal carcinoma cell lines were treated with nobiletin for 24 h. We found that the inhibitory effect of nobiletin on cell proliferation was dose-dependent. When the nobiletin concentration was increased to 80 µM, the proliferative capacity of ACHN cells started to decrease, showing a cell viability value of 83.06 ± 3.88% (*P* < 0.05). At a concentration of 120 µM, viability was further reduced to 66.43 ± 0.45% (*P* < 0.05) (**Figure 1A**). The proliferative capacity of Caki-2 cells started to drop at a nobiletin concentration of 40 µM, with a viability value of 89.23 ± 1.10% (*P* < 0.05). When the nobiletin concentration reached 80 µM, viability was 67.36 ± 2.81% (*P* < 0.05) (**Figure 1B**).

Subsequently, ACHN and Caki-2 cells were treated with different nobiletin concentrations for 12, 24, and 48 h. The results showed that the proliferative capacity of both cell lines was inhibited by nobiletin in a time-dependent manner (**Figure 1C, D**). Finally, we chose 40 and 80 µM, and 80 and 120 µM, as the working nobiletin concentrations for Caki-2 and ACHN cells, respectively.

In the plate colony-formation assay, a total of 400 cells were added to each well of a six-well plate. Nobiletin was added after cell attachment, and the medium was replaced by fresh medium after 48 h, followed by continued culture for 2 weeks. We observed that the number of colonies in the control group was higher than in the nobiletin-treated groups (**Figure 1E–H**).

### Nobiletin Promoted Apoptosis in Renal Carcinoma Cells

**Figure 2:**
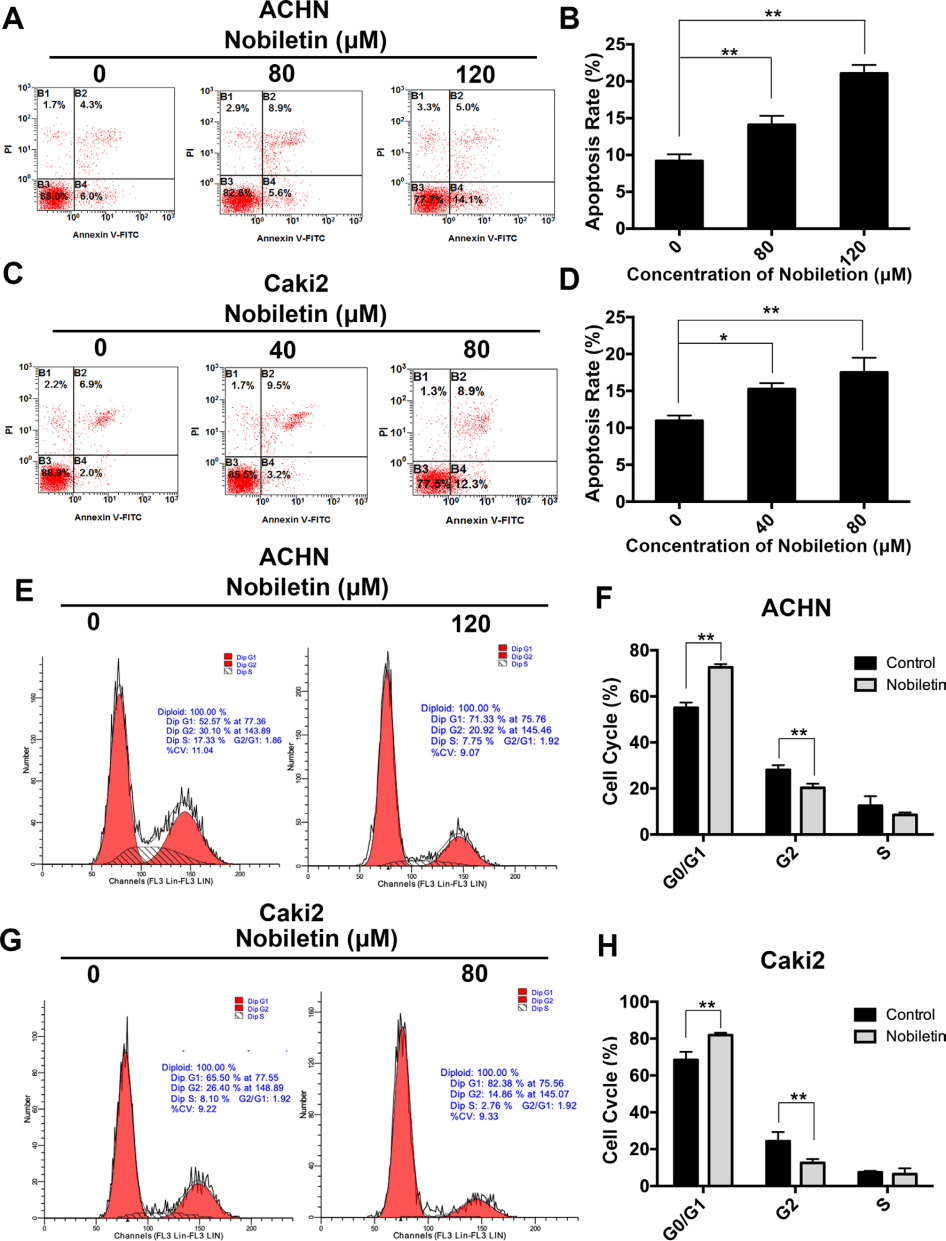
Nobiletin induces apoptosis and G0/G1 phase arrest in renal cancer cells. ACHN **(A)** and Caki-2 **(C)** cells were treated with different concentrations of nobiletin for 48 h, and then examined by flow cytometry for assessment of apoptosis. The apoptosis rates are shown in the graph **(B, D)**. ACHN **(E)** and Caki-2 **(G)** cells were treated with or without nobiletin for 48 h, and then examined by flow cytometry to analyze the cell cycle. The proportion of cells in each cell cycle stage is shown in the graph **(F, H)**. Data are presented as means ± SD. *P < 0.05, **P < 0.01, as compared with control.

After demonstrating that nobiletin suppressed the proliferative ability of renal carcinoma cells, we investigated its effects on apoptosis. Nobiletin was used at concentrations of 40 and 80 µM, and at 80 and 120 µM, to treat Caki-2 and ACHN cells for 48 h, respectively. Flow cytometric analysis was used to assess the apoptotic state of the cells by PI and FITC-annexin V double labeling. The apoptotic rate of the ACHN cells in the control, 80 µM nobiletin-, and 120 µM nobiletin-treated groups was 9.2 ± 0.89%, 14.1 ± 1.22%, and 21.06 ± 1.15%, respectively (**Figure 2A**). The apoptotic rates of ACHN cells treated with 80 and 120 µM nobiletin were significantly increased (*P* < 0.05) (**Figure 2B**). The apoptotic rates of the Caki-2 cells in the control, 40 µM nobiletin-, and 80 µM nobiletin-treated groups were 10.96 ± 0.70%, 15.26 ± 0.80%, and 17.53 ± 1.98%, respectively (**Figure 2C**). Moreover, the apoptotic rates of the Caki-2 cells treated with 40 and 80 µM nobiletin were significantly higher than that of the control (*P* < 0.05) (**Figure 2D**).

### Nobiletin Induced G0/G1 Cell Cycle Arrest in Renal Carcinoma Cells

Previous studies have shown that the antitumor effect of drugs depends predominantly on the promotion of apoptosis or cell cycle arrest at specific regulatory points. To investigate whether nobiletin has an effect on the cell cycle of renal carcinoma cells, we used flow cytometry in conjunction with PI staining. In the control group, the proportions of ACHN cells in the G0/G1, S, and G2/M phases were 55.01 ± 2.81%, 28.08 ± 1.99%, and 12.51 ± 4.19%, respectively. After treatment with nobiletin for 24 h, the corresponding proportions were 72.65 ± 1.30%, 20.33 ± 1.78%, and 8.57 ± 1.08% (**Figure 2E**). Thus, the proportion of ACHN cells in the G0/G1 phase increased significantly (*P* < 0.05) (**Figure 2F**). Similar results were also observed for Caki-2 cells (**Figure 2G, H**). These data likely explain why nobiletin can significantly inhibit the proliferation of renal carcinoma cells.

### Nobiletin Inhibited Invasion and Migration by Renal Carcinoma Cells

**Figure 3:**
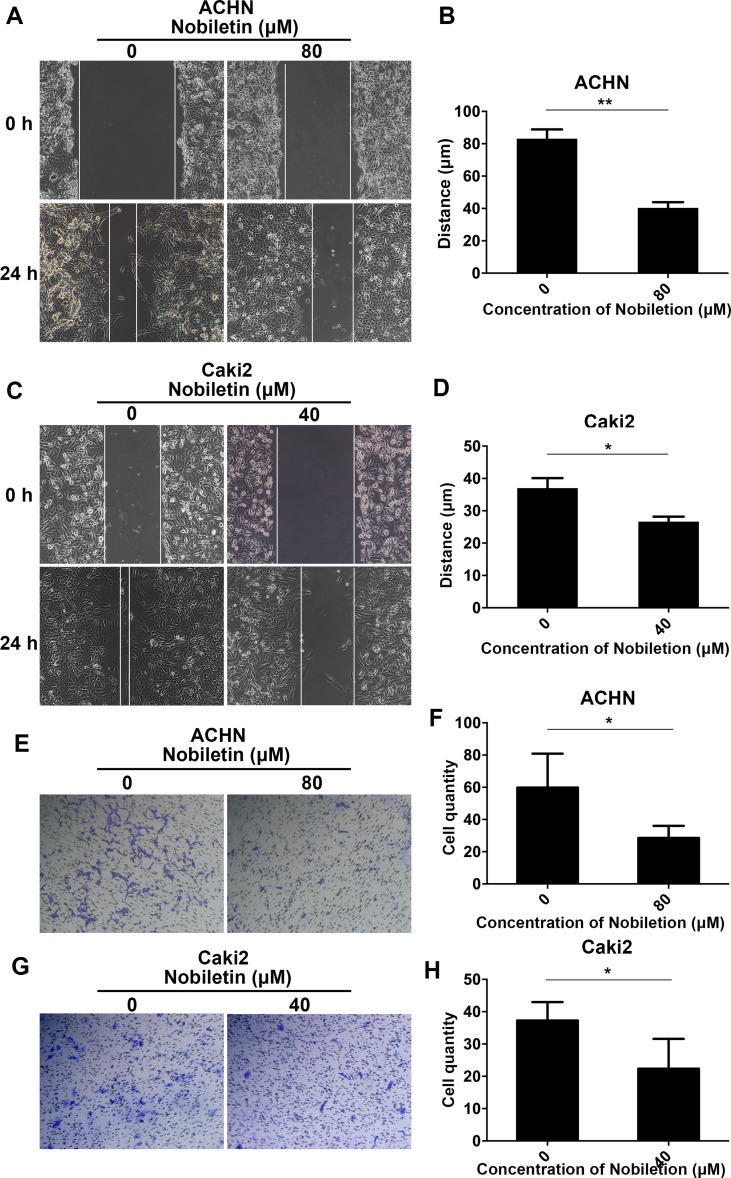
Nobiletin inhibits the migration and invasiveness of renal cancer cells. ACHN **(A)** and Caki-2 **(C)** cells were wounded and then treated with or without nobiletin for 24 h. Images were taken at 0 and 24 h (×100 magnification). The migration distance of ACHN **(B)** and Caki-2 **(D)** cells is shown in the graph. Invasion by ACHN **(E)** and Caki-2 **(G)** cells after 24 h. The number of invasive cells is shown **(F–H)**. Data are presented as means ± SD. *P < 0.05, **P < 0.01, as compared to control.

As a considerable number of studies have demonstrated that nobiletin exerts a significant inhibitory effect on cancer cell invasion and migration, we also investigated its ability to inhibit these two processes in renal carcinoma cells. We treated Caki-2 and ACHN cells with 40 and 80 µM nobiletin for 24 h, respectively. At these concentrations, the inhibitory effect of nobiletin on cell proliferation was extremely weak. In the scratch test, we observed that nobiletin significantly reduced the migration ability of renal carcinoma cells at the above-mentioned concentrations (**Figure 3A–D**). In the invasion assay, cell invasiveness was also significantly reduced (**Figure 3E–H**). Taken together, the results indicated that nobiletin exerts a significant inhibitory effect on the proliferation, invasion, and migration of renal carcinoma cells.

### Nobiletin Suppressed the Phosphorylation of SRC/AKT, STAT3, and YY1AP1 in Renal Carcinoma Cells

**Figure 4:**
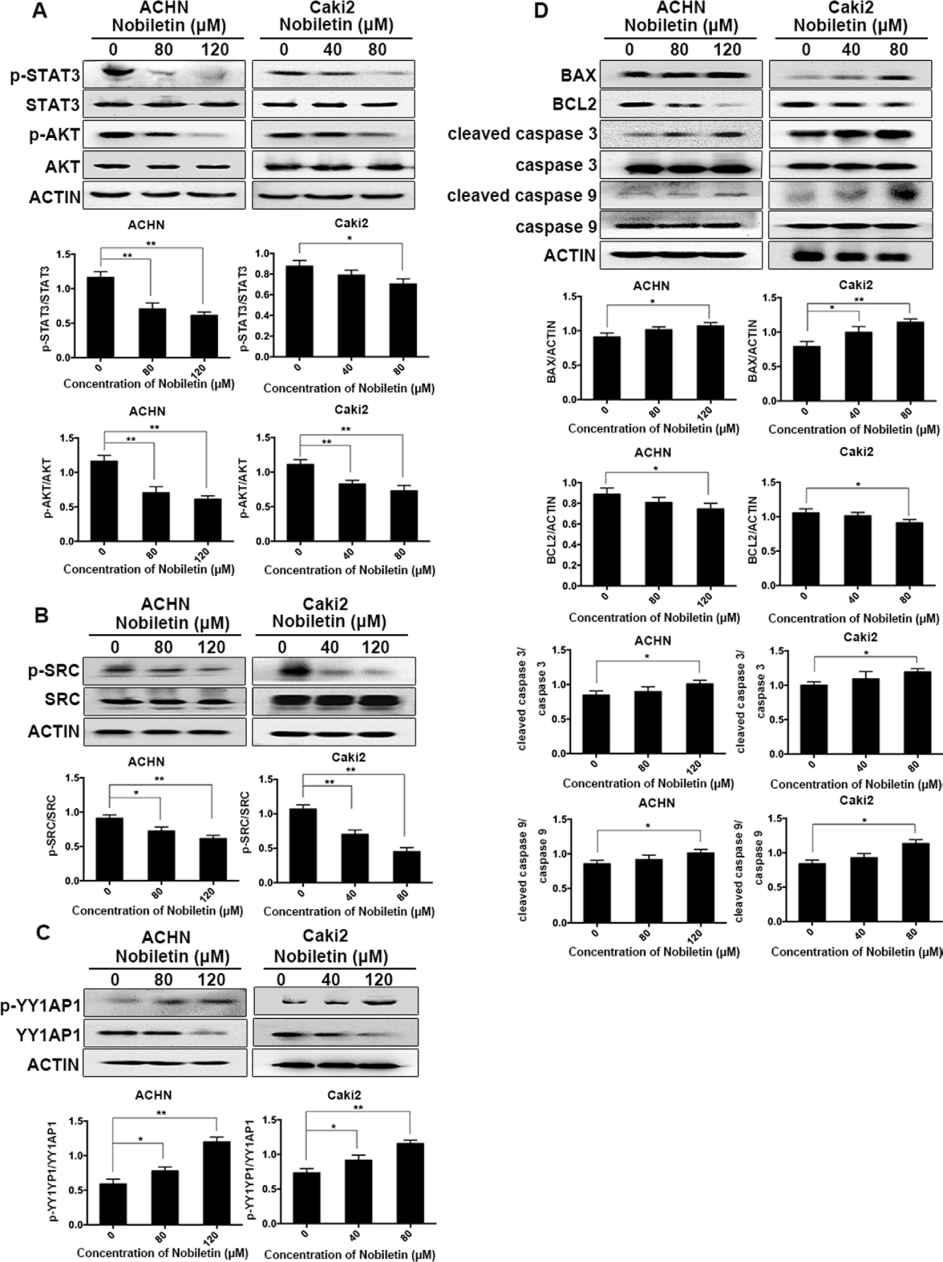
Nobiletin inhibits the SRC/AKT pathway, STAT3, and YY1AP1 activation, and induces apoptosis-related protein expression. ACHN and Caki-2 cells were treated with different concentrations of nobiletin (0, 80, or 120 µM for ACHN; 0, 40, and 80 µM for Caki-2) for 24 h **(A–C)** or 48 h **(D)**. The levels of phosphorylated AKT, phosphorylated STAT3, phosphorylated SRC, and the YY1AP1 protein were decreased, whereas the level of phosphorylated YY1AP1 was increased **(A–C)**. The levels of BAX, cleaved caspase 3, and cleaved caspase 9 were increased, whereas that of BCL2 was decreased **(D)**. Protein levels were examined by Western blot. Beta-actin was used as a control. Data are presented as means ± SD. *P < 0.05, **P < 0.01.

Western blotting was used to analyze the phosphorylation status of AKT in ACHN and Caki-2 cells after nobiletin treatment. Compared to the control group, AKT phosphorylation was significantly decreased in nobiletin-treated renal carcinoma cells (**Figure 4A**). We also investigated the changes in SRC phosphorylation in response to nobiletin treatment. The results showed that nobiletin significantly reduced the SRC phosphorylation level (**Figure 4B**).

In addition to AKT, STAT3 and YY1AP1 levels also share a close correlation with the proliferative ability of cells. We found that the phosphorylation levels of STAT3 in both ACHN and Caki-2 cells were decreased following nobiletin treatment (**Figure 4A**). Although YY1AP1 levels also decreased, the levels of phosphorylated YY1AP1 increased after nobiletin treatment (**Figure 4C**).

### Nobiletin Promoted the Expression of Apoptosis-Related Proteins

Since nobiletin could induce apoptosis, we used Western blot analysis to investigate changes in the levels of apoptosis-related proteins following nobiletin treatment to elucidate the mechanism involved in nobiletin-induced apoptosis. ACHN and Caki-2 cells were treated with 80 and 120 µM, and 40 and 80 µM nobiletin, respectively, for 48 h. The results showed that the levels of the proapoptotic proteins, cleaved caspase 3 and cleaved caspase 9, were increased compared to the control group. Furthermore, the expression of the antiapoptotic protein, BCL2, gradually decreased with increasing nobiletin concentrations. Conversely, the expression of the proapoptotic protein, BCL2 associated X (BAX), increased gradually with increasing nobiletin concentrations (**Figure 4D**).

### Nobiletin Suppressed the Nuclear Localization of STAT3 and YY1AP1

**Figure 5:**
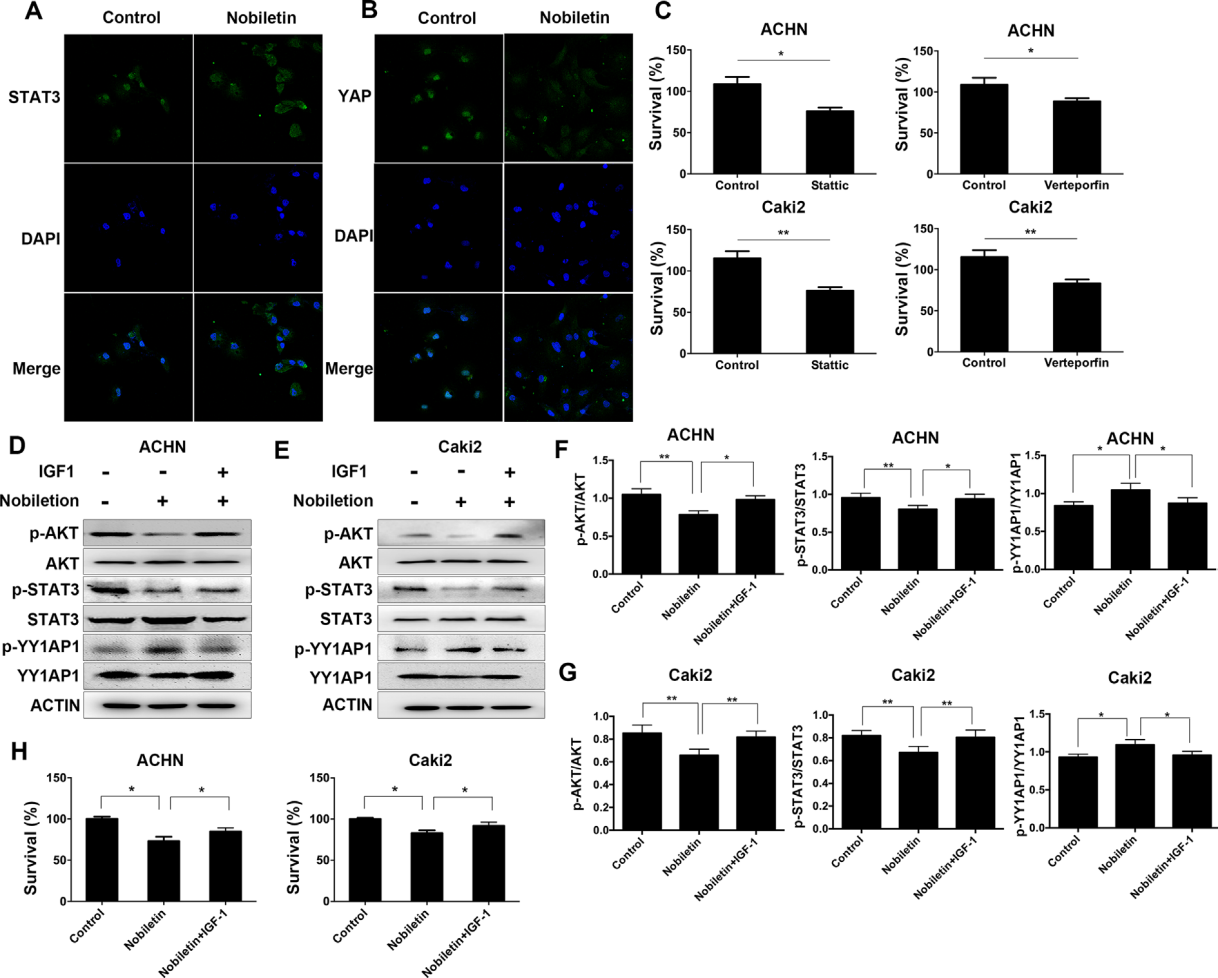
Nobiletin regulates STAT3 and YY1AP1 activation *via* activation of AKT. **(A, B)** Caki-2 cells were treated with (40 µM) or without nobiletin for 24 h, and then fixed and permeabilized. STAT3 and YY1AP1 were first stained with rabbit anti-STAT3 and anti-YAP primary antibodies, followed by FITC-conjugated secondary antibodies. The nucleus (blue) was stained with DAPI. The results showed that nobiletin treatment reduced the nuclear localization of STAT3 and YY1AP1. **(C)** ACHN and Caki-2 cells were treated with 4 µM Stattic and 4 µM Verteporfin, respectively. After 24 h, proliferation was evaluated by CCK-8 assay. **(D–G)** Renal cancer cells were treated with nobiletin (120 µM for ACHN, 80 µM for Caki-2) for 24 h and then IGF1 (200 ng/ml) was added for the final 2 h. The results showed that IGF1 reversed the effects of nobiletin on the function of these proteins. Beta-actin was used as a control. **(H)** Renal cancer cells were treated with nobiletin (120 µM for ACHN, 80 µM for Caki-2) for 24 h. IGF1 (200 ng/ml) was then added, and the cells were cultured for 24 h. Data are presented as means ± SD. *P < 0.05, **P < 0.01.

Since activated STAT3 and YY1AP1 can both enter the cell nucleus and induce the transcription of several specific genes, we used immunofluorescence and confocal microscopy to investigate the cellular localization of these proteins. The control group was Caki2 cells treated without nobiletin, and the experiment group was the Caki2 cells treated with nobiletin for 24 h. Compared to the control group, the nobiletin-treated group demonstrated reduced fluorescence intensity in the nucleus, indicating that translocation of STAT3 and YY1AP1 to the nucleus was affected. The results showed that nobiletin could inhibit the nuclear translocation of STAT3 and YY1AP1 (**Figure 5A, B**). Subsequently, the renal carcinoma cells were treated with Stattic (a STAT3 inhibitor) and Verteporfin (a YY1AP1 inhibitor) for 24 h. The results showed that cellular proliferation was inhibited. (**Figure 5C**).

### IGF1 Could Reverse the Effects of Nobiletin in Renal Carcinoma Cells

The control group consisted of cells without nobiletin treatment. One experimental group consisted of cells treated with nobiletin for 24 h, whereas the other experimental group contained cells treated with nobiletin for 24 h and IGF1 for the final 2 h; the levels of the related proteins were subsequently determined (**Figure 5D–G**). The levels of phosphorylated AKT, phosphorylated STAT3, and the YY1AP1 protein were decreased in the nobiletin-treated group compared to the control group. Furthermore, the level of phosphorylated YY1AP1 was increased in the nobiletin-treated group compared to the control group. Conversely, the levels of phosphorylated AKT, phosphorylated STAT3, and the YY1AP1 protein were increased in the group treated with nobiletin and IGF1 compared with the nobiletin only-treated group, whereas the level of phosphorylated YY1AP1 was decreased in the nobiletin- and IGF1-treated group compared to the nobiletin-treated group. These results indicated that IGF1 can reverse the effect of nobiletin on the function of these proteins.

To verify the antitumor effect of nobiletin *via* the regulation of AKT activation, renal cancer cells were treated with nobiletin for 48 h and IGF1 for the final 24 h, following which tumor viability was evaluated. The nobiletin- and IGF1-treated group exhibited significantly higher tumor viability than the group treated with nobiletin alone (**Figure 5H**). This suggested that activating AKT may reverse the antitumor effect of nobiletin and confirmed that nobiletin could inhibit tumor growth *via* the AKT pathway.

### Nobiletin Significantly Inhibited Tumor Cell Growth in Nude Mice

**Figure 6:**
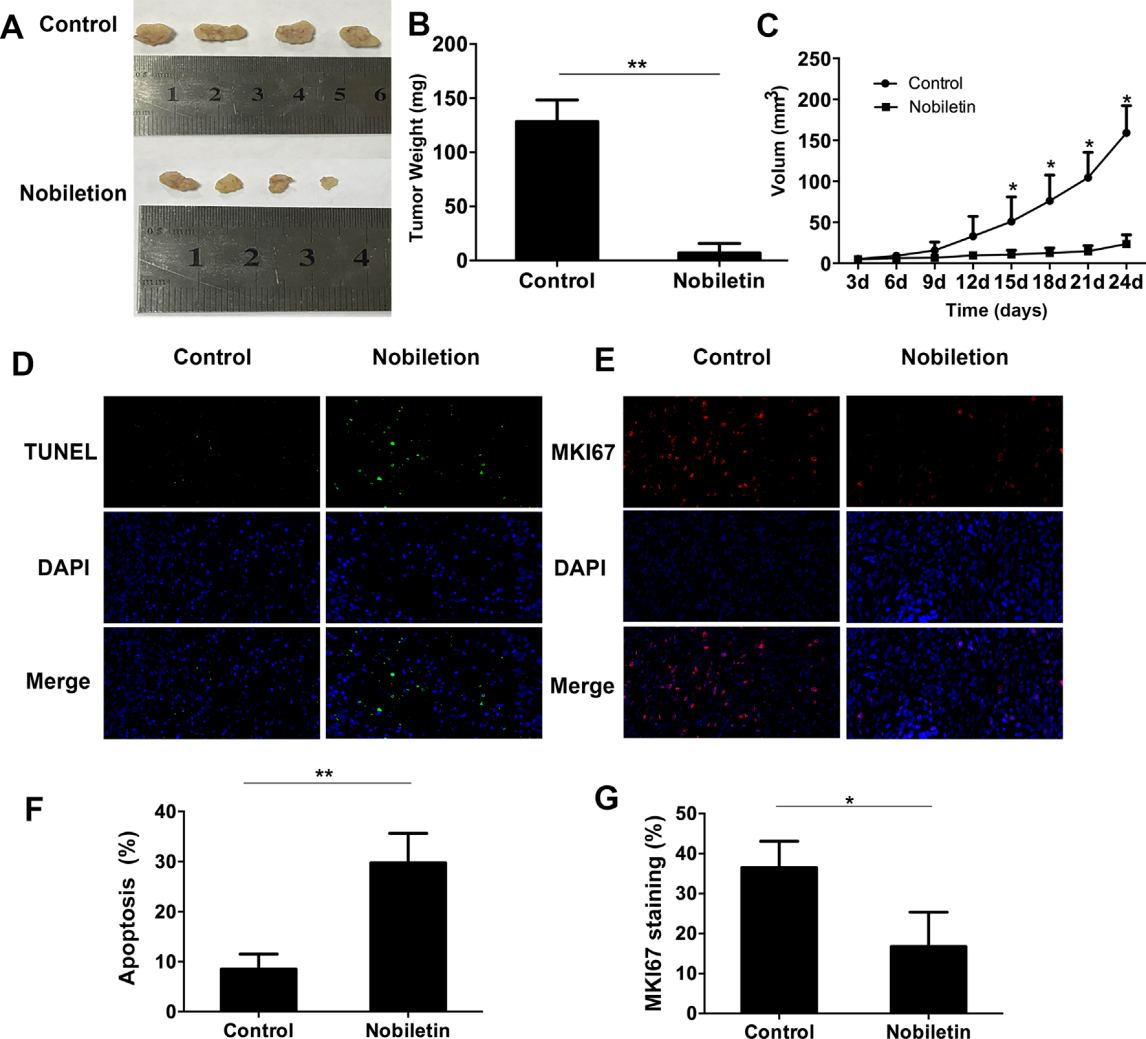
Nobiletin significantly inhibits xenografted tumor proliferation in nude mice (n = 4). ACHN cells were implanted subcutaneously into nude mice. The nude mice were then treated with or without nobiletin. Images were taken using a fluorescence microscope (×400 magnification). **(A, B)** The tumors were harvested and weighed. **(C)** Tumor volume was measured and compared. **(D, F)** TUNEL staining was conducted to determine the levels of apoptosis. **(E, G)** The expression levels of MKI67 were evaluated to determine cell proliferation. Data are presented as means ± SD. *P < 0.05, **P < 0.01, as compared to control.

To assess the tumor-suppressive effect of nobiletin *in vivo*, we used ACHN renal carcinoma cells to subcutaneously inoculate nude mice. The results showed that, starting at 15 days, the tumor volume in the nobiletin-treated group was markedly reduced compared to the control group, reaching a volume of 23.69 ± 11.04 mm^3^ at day 24. In the control group, the tumor volume was 159.10 ± 33.14 mm^3^ (*P* < 0.05) (**Figure 6A, C**). The tumor weight at day 24 was 6.98 ± 8.73 g in the nobiletin-treated group, significantly lower than that of the control group (128.40 ± 20.20 g) (*P* < 0.05) (**Figure 6B**).

We subsequently conducted fluorescent-immunohistochemical analysis of the xenografted tumor tissues to determine the expression levels of marker of proliferation KI67 (MKI67) (**Figure 6E**), as well as TUNEL staining to determine the degree of apoptosis (**Figure 6D**). The results showed that the expression level of MKI67 was significantly lower in the nobiletin-treated group (**Figure 6G**) and the degree of apoptosis was significantly higher (**Figure 6F**) than in the control.

To assess the toxicity of nobiletin, different doses of nobiletin (200 and 400 mg/kg·day^−1^) were administered to C57 mice *via* gastric lavage. The control group was administered the equivalent amount of physiological saline for 4 weeks. Compared to that before nobiletin treatment, we found no significant decrease in body weight after nobiletin treatment (**Supplementary Figure 1A**). Hematoxylin and eosin-stained sections showed no significant pathological changes in the heart, liver, kidney, spleen, or intestine of mice in the nobiletin-treated group compared to mice in the untreated group (**Supplementary Figure 1B**).

## Discussion

Several studies have reported the pharmacological characteristics of nobiletin and its inhibitory effect on different types of tumors. This compound is mainly extracted from plants of the *Citrus* genus, and its antitumor activities include inhibition of proliferation, cell cycle arrest, and promotion of apoptosis (Kim et al., 2016). In addition, nobiletin also targets and inhibits the metastatic characteristics of tumor cells. For example, Lee et al. reported that nobiletin decreased the expression of matrix metallopeptidase 2 (MMP2) and MMP9 in gastric cancer cells *via* the PTK2/PIK3CA pathway, thereby decreasing their invasiveness and migratory capacity (Lee et al., 2011). The two key aspects of antitumor activity, inhibition of proliferation and promotion of apoptosis, have also been reported for nobiletin. When evaluated against ovarian cancer, nobiletin reduced VEGF expression by inhibiting AKT activation, thereby reducing angiogenesis and inhibiting tumor proliferation (Chen et al., 2015). Using flow cytometry, Tang et al. (2007) showed that nobiletin exerted an apoptosis-promoting effect on tumor cells. To date, nobiletin has been reported to inhibit proliferation, invasion, and migration of gastric (Lien et al., 2016), prostate (Chen et al., 2014), liver (Shi et al., 2013), and ovarian cancer cells (Chen et al., 2015), among others. However, to the best of our knowledge, no studies have investigated its effects on kidney cancer prior to this study. Therefore, this is the first report on the inhibitory effect of nobiletin on the proliferation of renal carcinoma cells. Among these, ACHN cells, which are derived from a pleural effusion metastasis, are particularly malignant, and nobiletin exerted a time- and dose-dependent suppressive effect on these cells.

Compared to normal cells, tumor cells have a stronger proliferative capacity, which involves a series of signaling pathways. To investigate the underlying mechanism, we focused on the SRC/AKT signal transduction pathway. AKT activation was inhibited, which could further induce a growth arrest or apoptosis in the cells (Mundi et al., 2016). SRC proteins, which belong to the non-receptor tyrosine kinase family, promote mitosis in tumor cells during tumorigenesis and tumor development, and increase their adhesion and invasion capacity (Je et al., 2014). A focal point of antitumor therapy is the control of angiogenesis, in which SRC plays a key role through the upregulation of VEGF expression, consequently promoting new blood vessel formation. Our research showed that nobiletin can significantly inhibit SRC and AKT activation, as evidenced by the reduction in AKT and SRC phosphorylation with increasing nobiletin concentrations.

Activation of STAT3, an important transcription factor that regulates the expression of survivin, MMPs, and other proteins, has been observed in many tumors and is closely related to tumor cell survival and proliferation (Johnson et al., 2018). Some studies have reported that SRC overexpression can increase STAT3 activation, indicating the existence of a certain relationship between them (Silva, 2004). We demonstrated that SRC activation can be inhibited by nobiletin, suggesting that nobiletin may also inhibit STAT3 activation. The results showed that STAT3 phosphorylation decreased with increasing nobiletin concentrations.

Dysregulation of the Hippo signaling pathway can lead to pathogenesis, and a key downstream protein, YY1AP1, is generally overexpressed in tumor tissues. For example, Salcedo Allende et al. (2017) reported that, compared to chronic pancreatitis, YY1AP1 is significantly overexpressed in pancreatic ductal adenocarcinoma, and shows a correlation with hepatic metastasis. Tschaharganeh et al. (2013) reported that overexpress YY1AP1 in hepatoma carcinoma cell leading the proliferation. Consequently, we also investigated whether nobiletin could inhibit YY1AP1 expression. The results showed an increase in YY1AP1 phosphorylation with increasing nobiletin concentrations, with a concomitant reduction in YY1AP1 expression.

The promotion of tumor cell apoptosis by nobiletin is also a key factor in its antitumor activity. In the mitochondrial apoptosis pathway, BCL2 has an antiapoptotic role, whereas BAX promotes apoptosis following mitochondrial damage. Therefore, the induction and progression of apoptosis depend on the balance between these two proteins. Our results showed that nobiletin treatment decreased BCL2 expression in renal carcinoma cells, whereas that of BAX was increased. Furthermore, we found that nobiletin activated pro-caspase 3 and 9, thereby increasing the levels of cleaved caspase 3 and 9 in a dose-dependent manner. These results indicated that nobiletin exhibits an apoptosis-promoting effect.

In addition to the inhibition of tumor cell proliferation by nobiletin, we also demonstrated that nobiletin inhibited the invasiveness and migration capacity of renal carcinoma cells. Notably, this was also observed for the highly invasive ACHN cells, which are derived from pleural effusion metastasis. Earlier studies have shown that activating AKT inhibition can suppress cell migration (Marcial-Medina et al., 2019; Mete et al., 2019). Our own results indicated that nobiletin inhibits AKT activation, suggesting that this could be one of the mechanisms underlying the inhibitory effect of nobiletin on the invasion and migration of renal carcinoma cells.

We also investigated the functional relationship between AKT, STAT3, and YY1AP1. A comparison of organellar localization following gene activation indicated that STAT3 and YY1AP1 are primarily localized to the nucleus, whereas AKT remains in the cytoplasm. Li et al. (2017) discovered that AKT acts upstream of STAT3 in bladder cancer, activating the latter and promoting invasion and migration. Some studies also concluded that AKT acts upstream of STAT3 during apoptosis, exerting an important regulatory function (Wu et al., 2014). As a downstream molecule of the Hippo pathway, YY1AP1 is reportedly regulated by the PTK2/SRC/PIK3CA axis, increasing the adhesion force of fibronectin (Kim and Gumbiner, 2015). Strassburger et al. (2012) reported that IGF1 can increase YY1AP1 expression in liver cancer cells. Moreover, Li et al. found that AKT inhibitors can significantly increase the levels of phosphorylated YY1AP1 and proposed that AKT activation may promote YY1AP1 expression (Li et al., 2013). In our study, we observed that nobiletin significantly decreased the phosphorylation levels of AKT and STAT3, as well as YY1AP1 protein levels, and these three factors were closely related to cell proliferation. In addition, a mutual relationship has also been reported for AKT and YY1AP1. Consequently, we hypothesized that AKT may act as an upstream regulator of STAT3 and YY1AP1.

**Figure 7:**
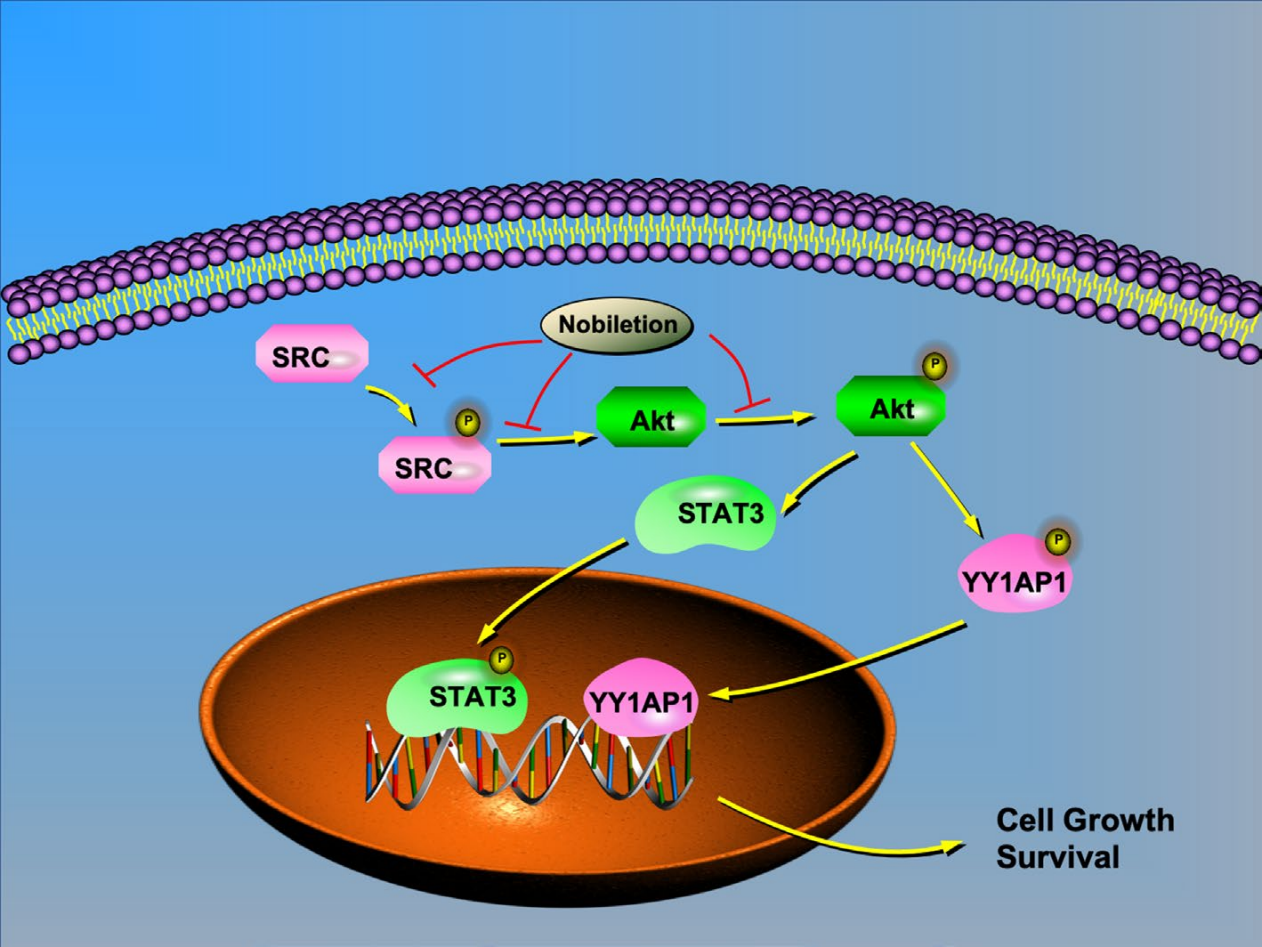
Schematic model depicting the possible mechanisms of nobiletin-mediated inhibition of renal cancer cell proliferation. AKT regulates the translocation of STAT3 and YY1AP1 to the nucleus. Nobiletin inhibits the SRC/AKT pathway and reduces STAT3 and YY1AP1 activation, thus contributing to the inhibition of cell proliferation.

Based on previous research and our study, we chose IGF1 as an AKT agonist and used it to stimulate AKT expression in renal carcinoma cells after nobiletin treatment. The results indicated that the suppression of AKT, STAT3, and YY1AP1 phosphorylation by nobiletin could be relieved by IGF1 treatment. Moreover, YY1AP1 phosphorylation was reduced following IGF1 treatment. Concomitantly, cells treated with both nobiletin and IGF1 showed higher tumor viability than cells treated with nobiletin alone, implying that activating AKT could reverse the antitumor effects of nobiletin, and confirmed that nobiletin could indeed inhibit tumor growth *via* the AKT pathway. This showed that AKT activation can promote the activation of STAT3 and YY1AP1, indicating that AKT acts upstream of both proteins. Accordingly, SRC can regulate the activation state of its downstream target, AKT. Hence, nobiletin may inhibit STAT3 and YY1AP1 activation by inhibiting the activation of AKT (**Figure 7**). Whether YY1AP1 and STAT3 activation provides a feedback that enhances the activation of the SRC/AKT pathway will be the subject of our future research.

*In vivo*, the tumor volume and weight in the nobiletin-treated group were markedly smaller and lower, respectively, compared with the control group. The level of apoptosis was higher in the nobiletin-treated group, indicating that the proliferative capacity was higher in the control group. *In vitro*, we demonstrated that nobiletin can inhibit SRC activation, which is associated with angiogenesis. This suggests that nobiletin can inhibit angiogenesis in tumors. We conducted fluorescent immunohistochemical analysis of the xenografted tumor tissues to determine the expression levels of platelet and endothelial cell adhesion molecule 1 (PECAM1), an indicator of vascular proliferation. However, there was no difference between the control and nobiletin-treated groups (data not shown). Therefore, we believe that the anticancer effect of nobiletin *in vivo* results mainly from the inhibition of tumor proliferation and promotion of apoptosis. We also assessed nobiletin toxicity in C57 mice. Following nobiletin treatment, the body weight of mice in the nobiletin-treated group was not significantly decreased compared to control. In addition, compared to the control group, no pathological changes were recorded in the heart, liver, kidney, spleen, and intestine in the nobiletin-treated group, indicating that normal tissues or organs can tolerate the adverse effects of nobiletin.

## Conclusion

In conclusion, this study showed that nobiletin could significantly suppress the proliferation, invasion, and migration of renal carcinoma cells and promote their apoptosis. The main mechanism involves the inhibition of the SRC/AKT pathway, which, in turn, suppresses the activation of the downstream molecules, STAT3 and YY1AP1.

## Ethics Statement

All animal experiments complied with ARRIVE guidelines and were carried out in strict accordance with the recommendations in the Guide for the Care and Use of Laboratory Animals of the National Institutes of Health (NIH Publication no. 8023, revised 1978). The protocol was approved by competent ethics committees at Institutes of Laboratory Animal, Fourth Military Medical University.

## Author Contributions

DW performed the mainly cell and animal experiment and wrote the article. GZ treated the sample and analyzed the data. DW and ZZ cultured the cell. YZ detected the apoptosis by flow cytometry. FY detected the cell cycle by flow cytometry. CP revised the manuscript. ZW and XL collected the data. FW and PM performed the migration assay. WZ performed the invasion assay. ZY performed the immunofluorescence. DZ performed the Western blot. ZL provided the technical guidance. JY designed the study.

## Funding

This work was supported by Scientific Innovative Project of Shaanxi Province (grant number 2012KTCL03-03) and Xijing Hospital subject booster plan translational medicine research projects (grant number XJZT13Z05).

## Conflict of Interest Statement

The authors declare that the research was conducted in the absence of any commercial or financial relationships that could be construed as a potential conflict of interest.

## Supplementary Material

Figure S1 Nobiletin was administered to C57 mice at different doses (200 mg/kg^−1^·day^−1^, and 400 mg/kg^−1^·day^−1^) (n = 4). The control group was administered the equivalent amount of physiological saline. Body weight in the different groups **(A)**. Hematoxylin and eosin staining of the heart, liver, kidney, spleen, and intestine in the different groups **(B)**.
