# Supplementary figures and images for: Nobiletin Inhibits Cell Viability via the SRC/AKT/STAT3/YY1AP1 Pathway in Human Renal Carcinoma Cells
Source: Front Pharmacol. 2019 Jul 9;10:690. doi: 10.3389/fphar.2019.00690 (PMC6635658; doi:10.3389/fphar.2019.00690)

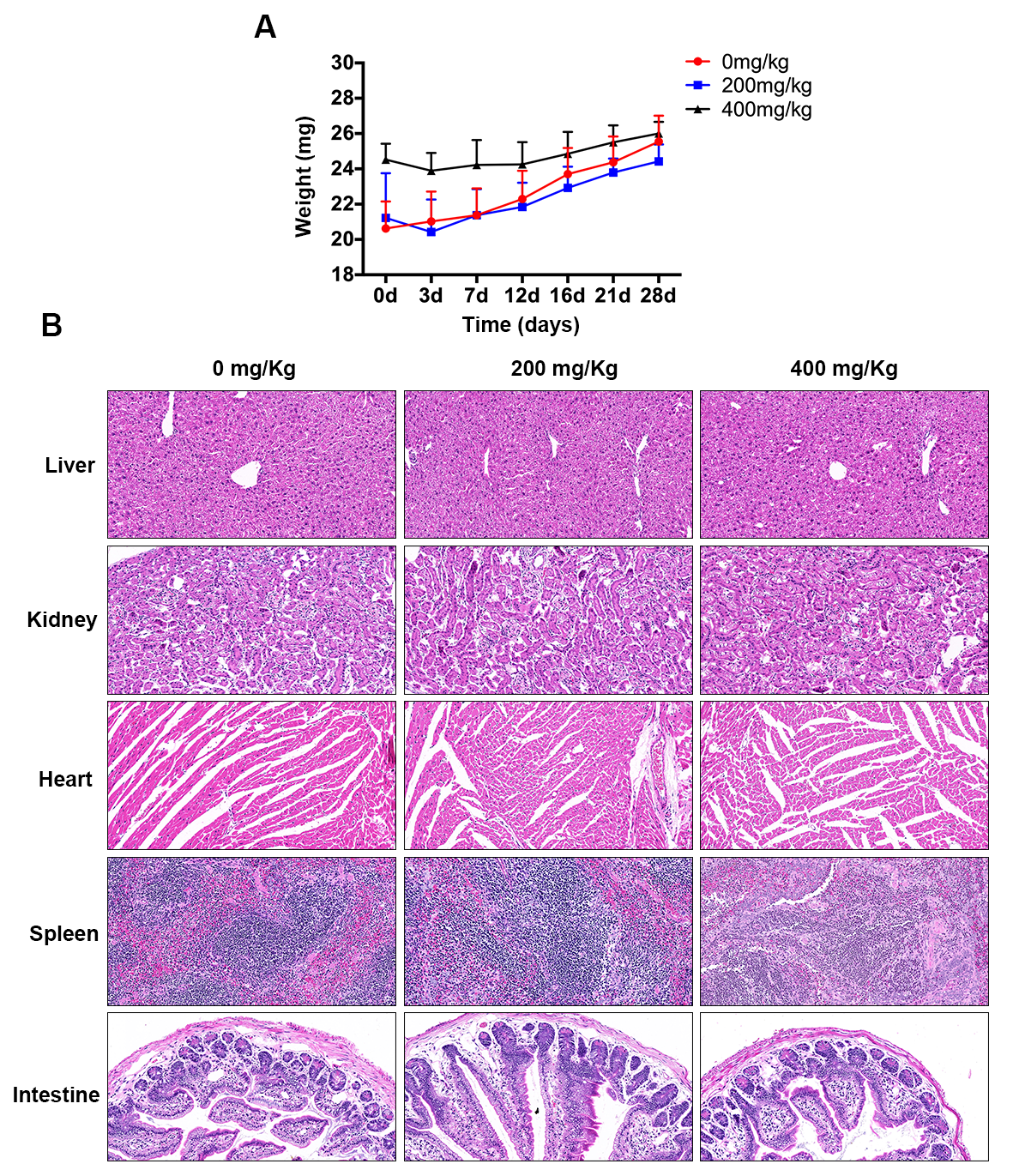

Supplement: Figure S1 — Nobiletin was administered to C57 mice at different doses (200 mg/kg−1·day−1, and 400 mg/kg−1·day−1) (n = 4). The control group was administered the equivalent amount of physiological saline. Body weight in the different groups (A). Hematoxylin and eosin staining of the heart, liver, kidney, spleen, and intestine in the different groups (B). [file Image_1.tif]
